# Clinical characteristics and health‐care resource utilization in patients with generalized pustular psoriasis using real‐world evidence from the Japanese Medical Data Center database

**DOI:** 10.1111/1346-8138.16084

**Published:** 2021-08-13

**Authors:** Yukari Okubo, Nirali Kotowsky, Ran Gao, Kumiko Saito, Akimichi Morita

**Affiliations:** ^1^ Department of Dermatology Tokyo Medical University Hospital Tokyo Japan; ^2^ Boehringer Ingelheim Pharmaceuticals Inc Ridgefield Connecticut USA; ^3^ Nippon Boehringer Ingelheim Co., Ltd Tokyo Japan; ^4^ Department of Geriatric and Environmental Dermatology Nagoya City University Graduate School of Medical Sciences Nagoya Japan

**Keywords:** comorbidity, disease burden, generalized pustular psoriasis, rare disease, real‐world evidence

## Abstract

Little is known about the disease burden, health‐care resource utilization (HCRU), or treatment of patients with generalized pustular psoriasis (GPP) in Japan. This retrospective cohort study used data from the Japanese Medical Data Center database to compare the demographics, comorbidities, and medication use of patients with GPP and plaque psoriasis and estimate their all‐cause HCRU. The patient selection period was from January 1, 2015 to December 31, 2019, and patients must have had at least one confirmed inpatient claim or outpatient claim for GPP or plaque psoriasis. During the 12‐month follow‐up period, 110 patients with GPP and 20,254 patients with plaque psoriasis were identified. An age‐ and sex‐matched (4:1) comparator control cohort, including members of the general population without a diagnosis of psoriasis (but allowing for a diagnosis of psoriatic arthritis), GPP, or palmoplantar pustulosis, was used. The most prevalent comorbidities in patients with GPP included allergic rhinoconjunctivitis, hypertension, and peptic ulcer disease. Patients with GPP were more likely to experience more comorbidities than those with plaque psoriasis, including asthma, chronic obstructive pulmonary disease, interstitial pneumonia, hyperuricemia and gout, tonsillitis, psoriatic arthritis, other psoriasis, and osteoporosis. Patients with GPP were more likely to be treated with a combination therapy than those with plaque psoriasis (65.5% vs 21.7%, respectively) and less likely to be treated with a topical medication alone (20.9% vs 50.8%). Patients with GPP had more outpatient visits than patients in the plaque psoriasis or matched control cohorts (mean [standard deviation], 14.8 [8.3] vs 11.0 [7.6] and 7.8 [7.2], respectively). They were also more likely to require inpatient hospitalization (24.5% vs 6.4% and 5.0%, respectively). Despite study limitations, patients with GPP in Japan were found to have a higher disease burden, including presence of comorbidities and medication use, than those with plaque psoriasis.

## 1 INTRODUCTION

Generalized pustular psoriasis (GPP) is a rare, intractable, potentially life‐threatening neutrophilic skin disease characterized by widespread diffuse, erythematous patches with visible pustules.^1^, ^2^, ^3^, ^4^

Historically, GPP has been classed as a subtype of psoriasis;^5^ it has only recently been established as a phenotypically and genetically separate disease entity to plaque psoriasis.^3^, ^4^, ^6^, ^7^ Due to its rarity, the clinical course of GPP is not well reported,^8^ and there are limited data on disease management and patient experiences of living with GPP compared with plaque psoriasis. In contrast to GPP, plaque psoriasis is the most common subtype of psoriasis. Plaque psoriasis is an inflammatory skin disease that is characterized by patches of erythematous scaly, papular, or plaque skin that may be painful or tender.^9^ It is also associated with comorbidities, including psoriatic arthritis, cardiometabolic diseases, and depression.^10^ For mild plaque psoriasis, treatments usually include topical medications, with biologics (specifically tumor necrosis factor [TNF] inhibitors) used as first‐line treatment for more severe cases of psoriasis.^10^ First‐line treatments for GPP usually include similar therapies, despite GPP being genetically and phenotypically distinct from plaque psoriasis, representing a potential unmet need for this patient population.^3^

There are limited data on the disease burden, health‐care resource utilization (HCRU), and treatment of patients with GPP in Japan. Understanding the disease burden in this population, including the associated comorbidities, is important from both the patient and health‐care system perspective. This will aid in the understanding of the different needs of patients with different skin diseases, and allow insights into how patients with different diagnoses are treated. Because GPP is a rare disease affecting a relatively small proportion of the Japanese population, there are limited data pertaining to the economic burden of the disease on the Japanese health‐care system. The potential major economic impact of rare diseases on health‐care systems has previously been reported,^11^ and understanding the HCRU and associated costs of managing GPP will be important to understand the impact of HCRU on health‐care budgets.

Here, we describe the characteristics and HCRU of employed patients with GPP in Japan using data from the Japanese Medical Data Center (JMDC) database, a commercially available claims database in Japan. The JMDC database is a real‐world, epidemiological receipt database that accumulates receipts from multiple health insurance associations submitted from inpatient, outpatient, dispensing pharmacy settings, and hospitals that use the Japanese diagnostic procedure combination payment system, as well as health check‐up data. All claims received are collected by the insurer. Patients enrolled in the JMDC database are employed individuals, and their dependents, from relatively large companies who are commercially insured, representing approximately 7.3 million patients in Japan.^12^ However, there are insufficient data for the population aged ≥65 years, and the data for those aged >75 years are also lacking.

Outcome measures in this study included comorbidities, dermatological medication use, concomitant medication use, and all‐cause HCRU and health‐care costs during the 12‐month follow‐up period.

## 2 METHODS

This retrospective cohort study used data on the characteristics (including demographics, comorbidities, medication use, and HCRU) of patients with GPP and plaque psoriasis from the JMDC database, an administrative claims database that holds accumulated prescription receipts (inpatient, outpatient, and dispensing) and health check‐up data from multiple health insurance associations.

**FIGURE 1:**
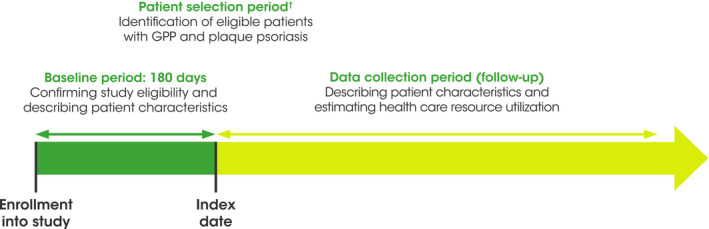
Study design. ^†^Patients were enrolled into a cohort when they fulfilled all study criteria (index date). GPP, generalized pustular psoriasis

The patient selection period was from January 1, 2015 to December 31, 2019, and patients were enrolled into the study at any time during this period once they fulfilled the study criteria, with a minimum baseline period of 6 months of continuous enrollment preceding the index date. The index date was the date of the first inpatient or outpatient claim with a confirmed diagnosis code of L40.0 (plaque psoriasis) or L40.1 (GPP), respectively. A data collection follow‐up period of 12 months started on the index date (Figure 1).

The International Classification of Diseases, 10th revision (ICD‐10 as dictated by the World Health Organization) codes and standard disease codes (named by the Japanese Ministry of Health, Labor, and Welfare) were used to identify patients with GPP (ICD‐10 code L40.1) and patients with plaque psoriasis (ICD‐10 L40.0). Patients included in each of these cohorts had at least one inpatient or outpatient confirmed claim code, according to standard criteria for identifying patients in claims databases. The third cohort was an age‐ and sex‐matched (4:1) comparator control cohort, matched to patients with GPP (denoted throughout as the “matched control cohort”). This cohort included members of the general population without a diagnosis of psoriasis (excluding ICD‐10 codes L40.0, L40.2, L40.4, L40.8, or L40.9), GPP (ICD‐10 code L40.1), or palmoplantar pustulosis (ICD10 code L40.3); however, inclusion criteria allowed for a diagnosis of psoriatic arthritis (L40.5). Patients with GPP and concomitant plaque psoriasis were included in the GPP cohort.

Only patients with a confirmed diagnosis (i.e., with one inpatient or outpatient ICD‐10 diagnosis code) of GPP or plaque psoriasis and at least 6 months of continuous enrollment prior to the index date were eligible for inclusion. All patients in the GPP and plaque psoriasis cohorts must have had 12 months of continuous enrollment post the index date to be included in the 12‐month follow‐up analyses. ICD‐10 and standard disease codes were also used to identify comorbidities.

All analyses were conducted using the Instant Health Data platform (Panalgo) and R software, version 3.5.2 (R Foundation for Statistical Computing). No comparative statistical analysis was undertaken; descriptive analyses were used throughout. This included the mean and standard deviation (SD), median and interquartile range for continuous variables, and frequencies and percentages for categorical variables. For HCRU analyses, all‐cause visits were included. All comorbidities and medications analyzed can be found in Tables S1–S4.

This study was approved by the ethics committee of the Yoyogi Mental Clinic (approval no. NBI207‐1). The study was also conducted in accordance with the Declaration of Helsinki.^13^

## 3 RESULTS

### 3.1 Patient demographics and baseline characteristics

**FIGURE 2:**
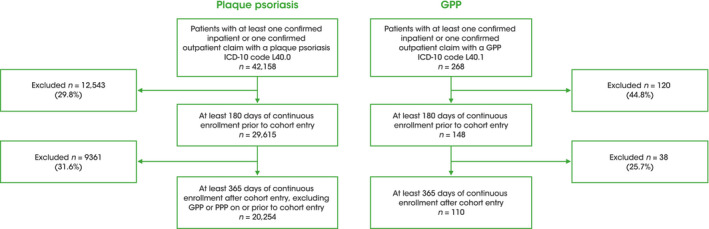
Study population. GPP, generalized pustular psoriasis; ICD‐10, International Classification of Diseases 10th revision; PPP, palmoplantar pustulosis

A total of 148 patients with GPP, 28,129 patients with plaque psoriasis, and 586 individuals in the matched control cohort were identified as having the required 6‐month baseline period preceding their index date. During the 12‐month follow‐up period, 110 patients with GPP, 20,254 patients with plaque psoriasis, and 436 individuals in the matched control cohort were identified and included in the analysis (Figure 2). The majority of patients across all three cohorts were male (GPP, 61.5%; plaque psoriasis, 60.8%; matched control, 61.8%) (Table 1) and the mean age of patients in the GPP, plaque psoriasis, and matched control cohorts was 45.0, 42.6, and 44.7 years, respectively. Patients with GPP were more likely to visit larger hospitals (≥20 beds, public or non‐public) than patients with plaque psoriasis (39.2% vs 12.5%) as well as university hospitals (13.5% vs 2.9%), whereas patients with plaque psoriasis were more likely to visit a smaller clinic (≤19 beds) than patients with GPP (83.2% vs 46.6%) (Table 1).

**TABLE 1:** Baseline characteristics

Demographics	GPP (n = 148)	Plaque psoriasis (n = 28,129)	Matched control cohort (n = 586)
Sex, male, n (%)	91 (61.5)	17,098 (60.8)	362 (61.8)
Age, years, mean (SD)	45.0 (16.6)	42.6 (16.2)	44.7 (16.4)
<18, n (%)	12 (8.1)	2892 (10.3)	48 (8.2)
18–64, n (%)	122 (82.4)	23,781 (84.5)	488 (83.3)
≥65, n (%)	14 (9.5)	1456 (5.2)	50 (8.5)
Insured, n (%)	100 (67.6)	18,792 (66.8)	403 (68.8)
Dependent, n (%)	48 (32.4)	9337 (33.2)	183 (31.2)
Type of hospital, n (%)			
Clinic (≤19 beds)	69 (46.6)	23,388 (83.2)	350 (59.7)
Other hospital^a^ (≥20 beds)	37 (25.0)	2692 (9.6)	70 (12.0)
Public hospital (≥20 beds)	21 (14.2)	830 (2.3)	11 (1.9)
University hospital	20 (13.5)	828 (2.9)	11 (1.9)
Unknown	N/A	4 (<0.1)	N/A
BMI among patients with at least one record, mean (SD), kg/m^2^	23.6 (3.9)^b^	23.8 (3.9)^b^	23.2 (3.5)

Abbreviations: BMI, body mass index; GPP, generalized pustular psoriasis; N/A, not applicable (data not available); SD, standard deviation.

^a^ Other hospital in a non‐public setting.

^b^ Missing patients are not included in these analyses.

### 3.2 Comorbidities during the 12‐month follow‐up period

**FIGURE 3:**
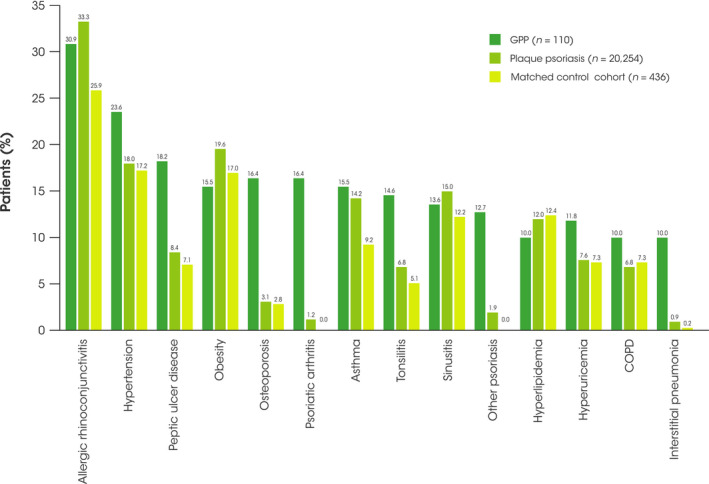
Proportions of patients with the most common comorbidities during the 12‐month follow‐up period. COPD, chronic obstructive pulmonary disease; GPP, generalized pustular psoriasis

The most prevalent comorbidities in patients with GPP included allergic rhinoconjunctivitis, hypertension, peptic ulcer disease, obesity, psoriatic arthritis, asthma, tonsillitis, sinusitis, other psoriasis, osteoporosis, hyperlipidemia, hyperuricemia or gout, chronic obstructive pulmonary disease (COPD), and interstitial pneumonia. In contrast to published literature,^14^ thyroid disorders were not among the most commonly reported comorbidities in those with GPP, despite being slightly more prevalent in those with GPP compared with those with plaque psoriasis and the matched cohort (4.6% vs 2.9% and 2.1%, respectively). During the 12‐month follow‐up period, patients with GPP were more likely to experience the majority of these comorbidities than those with plaque psoriasis and individuals in the matched control cohort; however, hyperlipidemia was more commonly diagnosed in both the plaque psoriasis and matched control cohorts compared with the GPP cohort (Figure 3). Obesity was more common among individuals in the matched control cohort than in patients with GPP or plaque psoriasis; however, not all patients in each cohort had a measure for body mass index, which may influence this finding.

### 3.3 Dermatological medication use during the 12‐month follow‐up period

Patients with GPP were more likely to receive treatment for their disease during the 12‐month follow‐up period than patients with plaque psoriasis (96.4% vs 78.5%, respectively) (Table 2). Patients with GPP were also more likely to be treated with a biologic monotherapy than patients with plaque psoriasis (1.8% vs 0.2%, respectively), and were also more likely than patients with plaque psoriasis to be treated with non‐biologic systemic therapy alone (8.2% vs 5.9%) (Table 2). Of the patients who received systemic therapies, more patients with GPP were treated with combination therapies (topical steroids + non‐biologic systemics; topical steroids + biologics; topical steroids + non‐biologic systemics + biologics; and biologic + non‐biologic systemic) than those with plaque psoriasis (65.5% vs 21.7%, respectively), and patients with GPP were less likely to be treated with a topical steroid alone compared with patients with plaque psoriasis (20.9% vs 50.8%) (Table 2).

**TABLE 2:** Systemic drug use during the 12‐month follow‐up period on or after the qualifying claim date

Treatment, n (%)	GPP (n = 110)	Plaque psoriasis (n = 20,254)
Biologic systemics	24 (21.8)	297 (1.5)
Biologics only	2 (1.8)	39 (0.2)
Topical steroids + biologics	4 (3.6)	95 (0.5)
Non‐biologic + biologic systemics	5 (4.5)	43 (0.2)
Topical steroids + non‐biologic systemics + biologic	13 (11.8)	120 (0.6)
Non‐biologic systemics	59 (53.6)	5320 (26.3)
Non‐biologic systemics only	9 (8.2)	1189 (5.9)
Topical steroids + non‐biologic systemics	50 (45.5)	4131 (20.4)
No systemic treatment	27 (24.5)	14,637 (72.3)
Topical steroids only	23 (20.9)	10,288 (50.8)
No treatment (topical steroids, non‐biologic systemics, and biologics)	4 (3.6)	4349 (21.5)

Note

The above groups are mutually exclusive.

Abbreviation: GPP, generalized pustular psoriasis.

**FIGURE 4:**
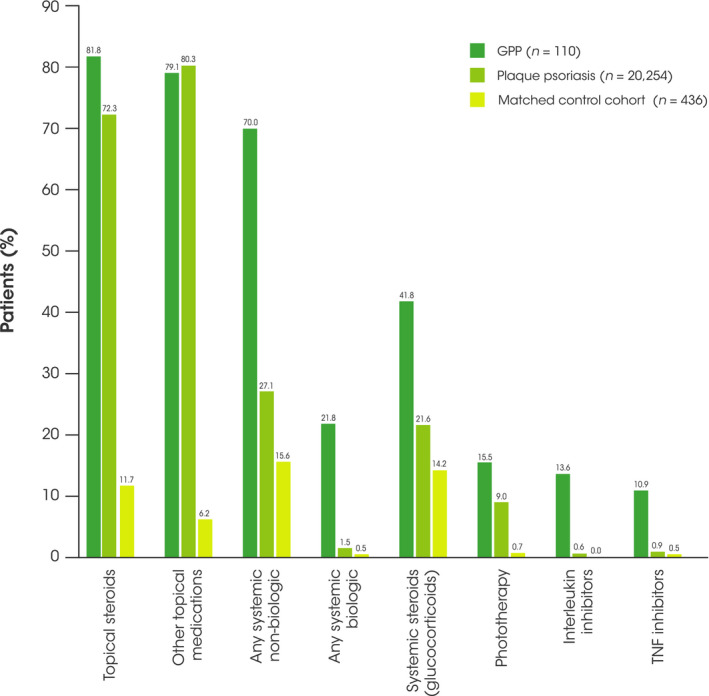
Medication use in patients with GPP and plaque psoriasis during the 12‐month follow‐up period. GPP, generalized pustular psoriasis; TNF, tumor necrosis factor

Of the non‐biologic therapies, the most commonly prescribed medications in patients with GPP were etretinate and cyclosporin, which were both more commonly prescribed in patients with GPP than in those with plaque psoriasis (etretinate, 17.3% vs 1.2%; cyclosporin, 28.2% vs 3.6%, respectively) (Figure 4). Of the biologic therapies, interleukin (IL) inhibitors were more commonly prescribed in patients with GPP than in those with plaque psoriasis (13.6% vs 0.6%, respectively), as were TNF inhibitors (10.9% vs 0.9%, respectively) (Figure 4). T‐cell inhibitors were not commonly prescribed in any of the cohorts (<0.1% for all). Of the topical medications, tacrolimus was received by 9.1% of patients with GPP, compared with 5.4% with plaque psoriasis. This is in contrast with systemic tacrolimus, which was received by just 0.9% of patients with GPP and 0.2% of patients with plaque psoriasis. Vitamin D_3_ was received by 10.9% of patients with GPP compared with 1.2% of patients with plaque psoriasis.

### 3.4 Concomitant medication use during the 12‐month follow‐up period

**FIGURE 5:**
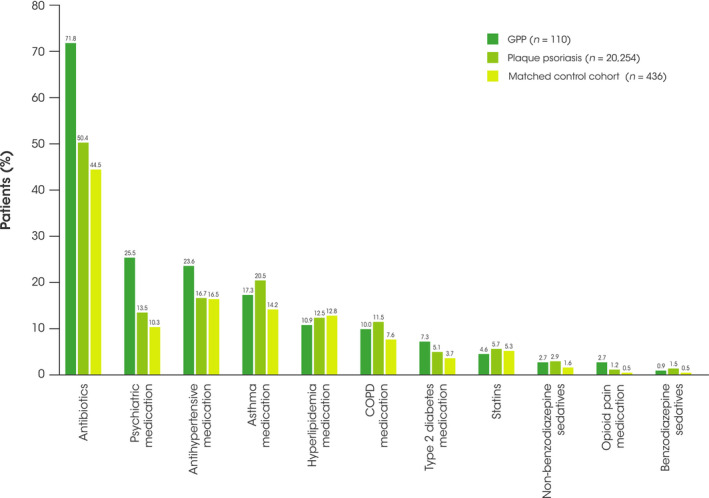
Most common concomitant medication use during the 12‐month follow‐up period. COPD, chronic obstructive pulmonary disease; GPP; generalized pustular psoriasis

During the 12‐month follow‐up period, patients with GPP generally received more frequent concomitant medication for their comorbidities than patients with plaque psoriasis and individuals in the matched control cohort (Figure 5). The most frequently prescribed medication across all cohorts was antibiotics; however, the proportion of patients with GPP requiring antibiotics was higher than the proportion of individuals in both the plaque psoriasis and control cohorts who required antibiotics (71.8% vs 50.4% and 44.5%, respectively).

Patients with GPP were nearly 2‐times as likely to be prescribed psychiatric medication than those with plaque psoriasis, and nearly 2.5‐times as likely than individuals in the matched control cohort. Patients with GPP were also more likely to receive antihypertensive medication, type 2 diabetes medication, and opioid pain medication than those with plaque psoriasis and individuals in the matched control cohort (though overall use of opioid pain medication was low in both GPP and plaque psoriasis cohorts at 2.7% vs 1.2%, respectively). Patients with plaque psoriasis and individuals in the matched control cohort were more likely to be prescribed statins, hyperlipidemia medication, asthma medication, and COPD medication than patients in the GPP cohort (Figure 5); however, these differences were small, and no large differences were observed between all three cohorts.

### 3.5 All‐cause HCRU during the 12‐month follow‐up period

Compared with patients with plaque psoriasis and individuals in the matched control cohort, the mean number of outpatient visits for patients with GPP was higher (mean [standard deviation [SD]], 14.8 [8.3] vs 11.0 [7.6] and 7.8 [7.2], respectively). Patients with GPP were also more likely to require inpatient hospitalization (24.5% vs 6.4% and 5.0%) (Table 3). In addition, the mean duration of hospitalization was between 2 and 4 days longer on average for patients in the GPP cohort than for patients with plaque psoriasis or individuals in the matched control cohort (Table 3).

**TABLE 3:** Overall all‐cause HCRU during the 12‐month follow‐up period

HCRU	GPP (n = 110)	Plaque psoriasis (n = 20,254)	Matched control cohort (n = 436)
All‐cause outpatient visits, n (%)	110 (100)	20,251 (>99.0%)	377 (86.5)
Mean (SD)	14.8 (8.3)	11.0 (7.6)	7.8 (7.2)
Median (IQR)	13.0 (10.0–18.0)	9.0 (5.0–15.0)	5.0 (2.0–12.0)
All‐cause inpatient visits, n (%)	27 (24.5)	1300 (6.4)	22 (5.0)
Inpatient visits >24 h, n (%)	27 (24.5)	1256 (6.2)	20 (4.6)
Mean (SD)	1.5 (1.1)	1.3 (0.8)	1.1 (0.2)
Median (IQR)	1.0 (1.0–2.0)	1.0 (1.0–1.0)	1.0 (1.0–1.0)
Duration of hospitalization >24 h, days			
Mean (SD)	12.0 (7.4)	10.0 (9.8)	8.7 (6.8)
Median (IQR)	10.0 (7.0–15.0)	7.0 (3.0–13.0)	8.0 (4.0–11.0)
Actual outpatient costs, ¥, ×10^3^			
Mean (SD)	632.9 (1054.2)	139.6 (384.5)	104.3 (325.7)
Median (IQR)	173.9 (93.9–509.4)	66.6 (30.9–132.0)	37.8 (16.2–93.3)
Actual inpatient costs, ¥, ×10^3^			
Mean (SD)	1492.7 (2838.7)	939.4 (1431.4)	505.7 (486.6)
Median (IQR)	586.6 (428.4–926.2)	478.6 (189.8–1057.8)	394.2 (112.0–794.8)
Actual pharmacy costs, ¥, ×10^3^			
Mean (SD)	285.9 (364.3)	89.8 (236.5)	68.5 (170.8)
Median (IQR)	141.3 (47.2–389.9)	38.1 (14.9–95.2)	17.0 (5.8–52.8)
Actual total costs, ¥, ×10^3^			
Mean (SD)	1274.8 (1843.2)	284.5 (686.0)	161.4 (434.3)
Median (IQR)	564.8 (200.6–1619.7)	118.1 (54.6–249.5)	44.7 (11.9–127.1)
Outpatient prescription costs, ¥, ×10^3^			
Mean (SD)	562.0 (1075.7)	59.8 (345.9)	36.7 (216.8)
Median (IQR)	25.4 (2.5–493.3)	3.4 (0.6–15.7)	2.4 (0.5–10.0)
Inpatient prescription costs, ¥, ×10^3^			
Mean (SD)	213.9 (640.9)	105.0 (456.5)	16.8 (20.7)
Median (IQR)	30.0 (8.8–89.3)	20.4 (5.7–45.9)	10.6 (2.0–24.6)
Pharmacy prescription costs, ¥, ×10^3^			
Mean (SD)	255.1 (353.1)	70.4 (228.4)	53.3 (161.3)
Median (IQR)	114.2 (31.2–326.8)	23.2 (8.0–67.1)	8.9 (2.2–34.9)
Total prescription costs, ¥, ×10^3^			
Mean (SD)	763.3 (1084.4)	117.1 (404.9)	57.4 (217.4)
Median (IQR)	249.9 (97.4–1045.4)	32.1 (11.9–87.7)	7.1 (0.7–30.8)

Notes

Prescription costs are calculated using standard drug price. Standard drug price is designated by Medical Remuneration in Japan and is renewed every 2 years. The limitation of using standard cost is the cost is not necessarily the reimbursed cost, and this cost will not vary between facilities in the same time for the same medication code.

Abbreviations: GPP, generalized pustular psoriasis; HCRU, health‐care resource utilization; IQR, interquartile range; SD, standard deviation.

Overall, patients with GPP had higher all‐cause total costs than patients with plaque psoriasis and individuals in the matched control cohort (mean [SD]: GPP, ¥1.27 million [1.84 million]; plaque psoriasis, ¥284.5K [686.0K]; matched control cohort, ¥161.4K [434.3K]) (Table 3). Stratification for prescription costs by settings showed that patients with GPP had higher prescription costs than patients with plaque psoriasis and individuals in the matched control cohort in outpatient, inpatient, and pharmacy settings (Table 3).

The most common reason for both outpatient and inpatient visits across the GPP cohort was psoriasis (diagnosis code L40) (Tables 4 and 5). Most patients with GPP and plaque psoriasis had outpatient visits with this claim code (98.2% vs 99.9%); while, more patients with GPP had inpatient visits with this claim code (66.7%) than patients with plaque psoriasis (13.7%). Other common reasons for inpatient visits for patients with GPP included other skin conditions (such as dermatitis and epidermal thickening), hypertension, gastrointestinal disorders (such as gastroesophageal reflux, gastritis and duodenitis, gastroenteritis and gastric ulcer), osteoporosis, and metabolic disorders (such as lipidemia and unspecified diabetes) (Table 4). Other common reasons for outpatient visits for patients with GPP included gastrointestinal disorders (such as those previously listed), skin disorders (including other dermatitis, atopic dermatitis, both of which were higher in the GPP cohort compared with the plaque psoriasis cohort, and epidermal thickening), respiratory disorders (such as bronchitis, allergic rhinitis, and pharyngitis), infections (including conjunctivitis and skin infections), metabolic disorders, and hypertension (Table 5). Unlike the proportions of patients with GPP having inpatient visits being higher for all conditions compared with patients with plaque psoriasis, the proportion of patients having outpatient visits was similar across all cohorts for some diagnoses, including psoriasis and epidermal thickening (other) (Table 5).

**TABLE 4:** Inpatient visits (based on the first three digits of the ICD‐10 code^a^) experienced by ≥8.0% of patients with GPP during the 12‐month follow‐up period

ICD‐10 diagnostic code	Diagnosis, n (%)	GPP (n = 110)	Matched control cohort (n = 436)	ICD‐10 diagnostic code	Diagnosis, n (%)	Plaque psoriasis (n = 20,254)
	Total number of patients with at least one inpatient diagnostic claim	(n = 27)	(n = 21)		Total number of patients with at least one inpatient diagnostic claim	(n = 1289)
L40	Psoriasis	18 (66.7)	0 (0.0)	I10	Essential (primary) hypertension	266 (20.6)
K21	Gastroesophageal reflux disease	6 (22.2)	3 (14.3)	K59	Other functional intestinal disorders	211 (16.4)
L30	Other dermatitis	6 (22.2)	0 (0.0)	E78	Disorders of lipoprotein metabolism and other lipidemias	192 (14.9)
L85	Other epidermal thickening	6 (22.2)	1 (4.8)	L40	Psoriasis	176 (13.7)
I10	Essential (primary) hypertension	5 (18.5)	4 (19.0)	G47	Sleep disorders	168 (13.0)
K29	Gastritis and duodenitis	5 (18.5)	3 (14.3)	K21	Gastroesophageal reflux	163 (12.6)
M81	Osteoporosis without pathologic fracture	5 (18.5)	0 (0.0)	E11	Type 2 diabetes	132 (10.2)
A09	Other gastroenteritis and colitis of infectious and unspecified origin	4 (14.8)	1 (4.8)	K29	Gastritis and duodenitis	131 (10.2)
K25	Gastric ulcer	4 (14.8)	2 (9.5)	K25	Gastric ulcer	122 (9.5)
T81	Complications of procedures, not otherwise classified	4 (14.8)	1 (4.8)	M54	Dorsalgia	104 (8.1)
D50	Iron‐deficiency anemia	3 (11.1)	2 (9.5)			
E14	Unspecified diabetes	3 (11.1)	2 (9.5)			
E78	Disorders of lipoprotein metabolism and other lipidemias	3 (11.1)	3 (14.3)			
J30	Vasomotor and allergic rhinitis	3 (11.1)	1 (4.8)			
L03	Cellulitis	3 (11.1)	0 (0.0)			
L08	Other local infections of the skin and subcutaneous tissue	3 (11.1)	0 (0.0)			
L51	Erythema multiforme	3 (11.1)	0 (0.0)			
R52	Pain, not otherwise classified	3 (11.1)	0 (0.0)			

Abbreviations: GPP, generalized pustular psoriasis; ICD‐10, International Classification of Diseases, 10th revision.

^a^ ICD‐10 codes within the Japanese Medical Data Center database align with World Health Organization disease classification.

**TABLE 5:** Outpatient visits (based on the first three digits of the ICD‐10 code^a^) experienced by ≥15.0% of patients with GPP during the 12‐month follow‐up period

ICD−10 diagnostic code	Diagnosis, n (%)	GPP (n = 110)	Matched control cohort (n = 436)	ICD‐10 diagnostic code	Diagnosis, n (%)	Plaque psoriasis n = 20,254
	Total number of patients with at least one outpatient diagnostic claim	(n = 110)	(n = 374)		Total number of patients with at least one outpatient diagnostic claim	n = 20,251
L40	Psoriasis	108 (98.2)	1 (0.3)	L40	Psoriasis	20,237 (99.9)
L30	Other dermatitis	63 (57.3)	50 (13.4)	L30	Other dermatitis	7922 (39.1)
L85	Other epidermal thickening	47 (42.7)	33 (8.8)	J30	Vasomotor and allergic rhinitis	7096 (35.0)
J30	Vasomotor and allergic rhinitis	35 (31.8)	120 (32.1)	L85	Other epidermal thickening	6894 (34.0)
K29	Gastritis and duodenitis	34 (30.9)	78 (20.9)	J06	Acute upper respiratory infections of multiple and unspecified origins	5974 (29.5)
M54	Dorsalgia	34 (30.9)	35 (9.4)	H52	Disorders of refraction and accommodation	5495 (27.1)
L08	Other local infections of the skin and subcutaneous tissue	33 (30.0)	11 (2.9)	K29	Gastritis and duodenitis	5085 (25.1)
J06	Acute upper respiratory infections of multiple and unspecified origins	31 (28.2)	119 (31.8)	J20	Acute bronchitis	5015 (24.8)
L20	Atopic dermatitis	31 (28.2)	11 (2.9)	H10	Conjunctivitis	4079 (20.1)
H52	Disorders of refraction and accommodation	28 (25.5)	100 (26.7)	E78	Disorders of lipoprotein metabolism and other lipidemias	3844 (19.0)
A09	Other gastroenteritis and colitis of infectious and unspecified origin	27 (24.5)	54 (14.4)	I10	Essential primary hypertension	3619 (17.9)
K21	Gastroesophageal reflux disease	27 (24.5)	34 (9.1)	J02	Acute pharyngitis	3435 (17.0)
E78	Disorders of lipoprotein metabolism and other lipidemias	25 (22.7)	86 (23.0)	B07	Viral warts	3180 (15.7)
I10	Essential (primary) hypertension	24 (21.8)	75 (20.1)	L20	Atopic dermatitis	3088 (15.2)
J20	Acute bronchitis	24 (21.8)	100 (26.7)			
J02	Acute pharyngitis	22 (20.0)	71 (19.0)			
L70	Acne	22 (20.0)	14 (3.7)			
B35	Dermatophytosis	21 (19.1)	12 (3.2)			
E14	Unspecified diabetes	20 (18.2)	32 (8.6)			
H10	Conjunctivitis	20 (18.2)	63 (16.8)			
K76	Other liver diseases	20 (18.2)	24 (6.4)			
K25	Gastric ulcer	19 (17.3)	31 (8.3)			
M06	Other rheumatoid arthritis	19 (17.3)	2 (0.5)			
M81	Osteoporosis without pathologic fracture	18 (16.4)	12 (3.2)			
G47	Sleep disorders	17 (15.5)	32 (8.6)			

Abbreviations: GPP, generalized pustular psoriasis; ICD‐10, International Classification of Diseases, 10th revision.

^a^ ICD‐10 codes within the Japanese Medical Data Center database align with World Health Organization disease classification.

Prescription claims data showed that the most common prescription claims in both GPP and plaque psoriasis cohorts were for potent corticosteroids (67.3% and 59.0%, respectively) and antipsoriatic drugs (59.1% and 76.2%). Other prescription claims that were highly reported in both cohorts were antihistamines for systemic use, other drugs for peptic ulcer and gastroesophageal reflux disease, and propionic acid derivatives (Table 6).

**TABLE 6:** Prescription claims based on the first five digits of the Anatomical Therapeutic Chemical Classification (WHO version) required by ≥15.0% of patients during the 12‐month follow‐up period

ATCC code	Medication class	GPP (n = 110) n (%)	Matched control cohort (n = 436) n (%)	ATCC code	Medication class	Plaque psoriasis (n = 20,254) n (%)
DO7AC	Corticosteroids, potent (group III)	74 (67.3)	34 (7.8)	DO5AX	Other antipsoriatics for topical use	15,441 (76.2)
DO5AX	Other antipsoriatics for topical use	65 (59.1)	1 (0.2)	DO7AC	Corticosteroids, potent (group III)	11,942 (59.0)
R06AX	Other antihistamines for systemic use	61 (55.5)	79 (18.1)	R06AX	Other antihistamines for systemic use	8125 (40.1)
J01DD	Third‐generation cephalosporins	54 (49.1)	105 (24.1)	R05CB	Mucolytics	6983 (34.5)
N01BB	Amides	53 (48.2)	64 (14.7)	M01AE	Propionic acid derivatives	6385 (31.5)
A02BX	Other drugs for peptic ulcer and GORD	52 (47.3)	86 (19.7)	A02BX	Other drugs for peptic ulcer and GORD	6001 (29.6)
M01AE	Propionic acid derivatives	51 (46.4)	100 (22.9)	N02BE	Anilides	5825 (28.8)
B05XA	Electrolyte solutions	46 (41.8)	73 (16.7)	J01DD	Third‐generation cephalosporins	5369 (26.5)
N02BE	Anilides	45 (40.9)	108 (24.8)	D07AB	Corticosteroids, moderately potent (group II)	5349 (26.4)
D07AD	Corticosteroids, very potent (group IV)	44 (40.0)	3 (0.7)	C05BA	Heparins or heparinoids for topical use	5234 (25.8)
C05BA	Heparins or heparinoids for topical use	42 (38.2)	28 (6.4)	D07AD	Corticosteroids, very potent (group IV)	4567 (22.5)
H02AB	Glucocorticoids	41 (37.3)	61 (14.0)	M02AA	Anti‐inflammatory preps, non­steroids topical use	4402 (21.7)
D07AB	Corticosteroids, moderately potent (group II)	35 (31.8)	25 (5.7)	N01BB	Amides	4214 (20.8)
V03AX	Other therapeutic products	34 (30.9)	65 (14.9)	B05XA	Electrolyte solutions	4191 (20.7)
L04AD	Calcineurin inhibitors	33 (30.0)	1 (0.2)	J01FA	Macrolides	4163 (20.6)
A07FA	Antidiarrheal micro‐organisms	31 (28.2)	62 (14.2)	V03AX	Other therapeutic products	3995 (19.7)
R06AX	Other antihistamines for systemic use	31 (28.2)	22 (5.0)	A07FA	Antidiarrheal micro‐organisms	3880 (19.2)
D02AX	Other emollients and protectives	30 (27.3)	9 (2.1)	B02AA	Amino acids	3808 (18.8)
R05CB	Mucolytics	29 (26.4)	140 (32.1)	H02AB	Glucocorticoids	3650 (18.0)
D07CC	Corticosteroids, potent, combination with antibiotics	27 (24.5)	24 (5.5)	D07CC	Corticosteroids, potent, combination with antibiotics	3480 (17.2)
B05BB	Electrolytes	26 (23.6)	38 (8.7)	R05DA	Opium alkaloids and derivatives	3243 (16.0)
R01AC	Antiallergic agents, excl. corticosteroids	24 (21.8)	22 (5.0)	J01MA	Fluoroquinolones	3238 (16.0)
A02BC	Proton‐pump inhibitors	23 (20.9)	28 (6.4)			
B02AA	Amino acids	23 (20.9)	70 (16.1)			
A02BA	H2‐receptor antagonists	22 (20.0)	20 (4.6)			

Abbreviations: ATCC, Anatomical Therapeutic Chemical Classification; GORD, gastroesophageal reflux disease; GPP, generalized pustular psoriasis; WHO, World Health Organization.

## 4 DISCUSSION

This study summarizes the clinical characteristics of patients with GPP in a real‐world setting in Japan and compares them with those of patients with plaque psoriasis and individuals in an age‐ and sex‐matched control (general population) cohort.

Across all cohorts in this study, prevalent comorbidities included allergic rhinoconjunctivitis, hypertension, and peptic ulcer disease. The comorbidities reported for patients with plaque psoriasis in this study are consistent with a previous study of patients in Japan with plaque psoriasis using the Japanese National Database of Health Insurance Claims.^15^ In both GPP and plaque psoriasis, defects in inflammatory mediators that are involved in allergic rhinoconjunctivitis, including pro‐inflammatory IL (specifically IL‐6) and TNF‐α,^16^, ^17^ also contribute to the manifestation of GPP and plaque psoriasis. However, approximately 44% of the overall population in Japan experience allergic rhinitis specifically;^18^ it is therefore possible that some patients with GPP visit the clinic for allergy treatment, not necessarily as a comorbidity of GPP. Peptic ulcer disease was also among the most prevalent comorbidities in patients with GPP and plaque psoriasis. Gastrointestinal disorders (specifically bleeding and perforation) may be triggered by continued use of oral steroids (specifically in patients who are hospitalized),^19^ such as those used to manage GPP; this may be confounded by the fact that peptic ulcer disease is associated with a number of comorbidities that are prevalent among patients with GPP, including COPD and type 2 diabetes.^20^ Prescriptions for peptic ulcer disease medications were among the highest claimed; however, treatments for gastrointestinal disorders were not among the most received medications by patients with GPP. Common diagnostic codes for inpatient visits often included those for gastrointestinal disorders; however, although the treatments received by patients during their hospital visits are recorded, data regarding surgeries and procedures are not evaluated in this study; therefore, more detail about how these disorders were treated is unknown. In this study, diagnostic codes were not linked to medications received; therefore, it is not possible to identify why certain medications were received.

Although the JMDC database does not distinguish patients who smoke from those who do not smoke, smoking may contribute to the increased prevalence of COPD in the GPP and plaque psoriasis populations. The Japanese Society for Psoriasis Research database reported that over a quarter (26.1%) of patients with all types of psoriasis are smokers.^21^ Another prevalent respiratory comorbidity was interstitial pneumonia. Interstitial lung disease has previously been associated with biologic use in patients with different types of psoriasis in Japan.^22^ The use of biologics may therefore contribute to the increased prevalence of interstitial pneumonia in patients with GPP in Japan; however, this association is not clear from this database. Steroids have been associated with development of osteoporosis, specifically after corticosteroid initiation.^23^ Given that corticosteroids were among the medications with the highest prescription claims in both GPP and plaque psoriasis cohorts, this may have contributed to increased prevalence of osteoporosis in these cohorts, given that prevalence was particularly high in this database, despite the cohort of patients being relatively young.

The greater use of psychiatric medication in patients with GPP compared with those with plaque psoriasis and individuals in the matched control cohort suggests that patients with GPP may experience greater emotional burden. However, psychiatric comorbidities were not among the most prevalent in patients with GPP. Diagnostic codes were not linked to medications; therefore, it is not possible to ascertain why certain medications were prescribed from this database.

Guidelines for the treatment of GPP in Japan (translated into English in 2018) recommend biologics for the treatment of GPP in Japan, including TNF inhibitors and IL inhibitors, as well as non‐biologic systemic therapies, such as etretinate, methotrexate, and cyclosporin for the symptoms of skin disease.^3^ In this study, only 21.8% patients with GPP received biologics (as monotherapies or as part of combination therapy with topical steroids or non‐biologic systemic therapies) during the 12‐month follow‐up period. This result demonstrates a limitation of the JMDC database, which covers employees (and their family members) who work at large companies in Japan. Patients with severe GPP who require biologics may be underrepresented as this database only includes patients who are employed and their families. It was more common for patients with GPP to receive combination medication. In comparison, most patients with plaque psoriasis in Japan were managed with topical medication only. The use of biologics in the plaque psoriasis population is similar to the use of biologics in a previous study of patients with plaque psoriasis (0.8%).^15^ While it is possible that there are many physicians in Japan who are not prescribing biologics, it is not possible to ascertain a reason for low use of biologics using this database, especially since the cost of biologics is likely to be covered by national health insurance. We show that 72.3% of patients with plaque psoriasis and 81.8% of patients with GPP received topical steroids, consistent with a previous claims database study of patients with psoriasis also identifying topical steroids as the most common treatments for psoriasis (81.4%).^15^

The differences in treatments for patients with GPP and those with plaque psoriasis suggests that disease severity is generally greater in patients with GPP; however, a limitation of this study is that it is not possible to infer the level of disease severity in the JMDC database. A degree of disease severity can, however, be inferred from the proportion of patients receiving systemic treatments. In this study, 75.5% of patients with GPP and 27.7% patients with plaque psoriasis received systemic treatments (biologic or non‐biologic), indicating that those with GPP were more likely to be experiencing severe disease than those with plaque psoriasis, who may have been more likely to display a more heterogeneous population of patients experiencing mild, moderate, and severe disease.

This analysis also suggests that patients with GPP have higher HCRU needs than those with plaque psoriasis and individuals in the matched control cohort. This includes overall health‐care costs, and reinforces how rare diseases can incur a high economic burden,^11^ suggesting that patients with GPP may experience prolonged complications of their disease.

The JMDC database is one of the largest claims databases in Japan for both industry and academic use;^24^ however, this study was not without limitations. The database includes data from receipts for prescriptions registered to an administrative claims database, and these data were collected for reimbursement purposes rather than scientific research. Whilst we can be certain that biologics administered intravenously. in hospital were received, it is more difficult to determine whether self‐administered medications were taken as prescribed after the prescription was filled. In addition, the study population is limited to individuals insured through specific health insurance associations, thus limiting the generalizability of the results to the general population in Japan. For example, this database only represents those who are employed in Japan (and their dependents), and this could impact the sex distribution of those with GPP in the JMDC database (where patients with GPP tended to be male, rather than female, as has been previously reported)^3^, ^5^ as males in Japan are more likely to be employed.^25^ In addition, this also means that there are very few patients captured who are older than 65 years of age, making it difficult to evaluate the disease burden in this age group. Additionally, the lack of a validation algorithm with positive predictive value to accurately identify patients with GPP, as well as a lack of consensus on the diagnostic criteria for a diagnosis of GPP specifically, presents a limitation in claims database studies; however, the stratification used in this study was based on standard procedures for retrospective claims database studies in Japan.

Given the employment status, overall disease severity of those in the database may be milder than that of the general population, which could include patients with disease so severe that they cannot work at all. A measure of work absenteeism, indicating a level of severity, can be inferred from the measure of inpatient visits, which was higher and of longer duration in patients with GPP than in those with plaque psoriasis; however, this may also be underrepresented because absenteeism may also include those who take days off sick from work, and these are not captured in the database. Finally, the JMDC database does not account for, or measure, the reasons underlying, specific treatment‐related decisions, such as treatment discontinuation, switching, and augmentation, and it does not fully cover, or measure, mortality data, including cause of death.

Despite the limitations stated above, the data included in this analysis represent the real‐life management of patients with GPP in Japan, and the results show that patients with GPP in Japan have a greater disease burden than those with plaque psoriasis. This study allowed for a detailed characterization of younger patients with GPP, which, to our knowledge, has not previously been performed.

## CONFLICTS OF INTEREST

Y. Okubo declares receiving research grants from Eisai, Maruho, and Shiseido Torii, and receives current consulting/advisory board agreements and/or speakers bureau fees and/or clinical trial funds from AbbVie, Amgen, Boehringer Ingelheim, Bristol Myers Squibb, Celgene, Eisai, Eli Lilly, Janssen Pharma, JIMRO, Kyowa Kirin, LEO Pharma, Maruho, Novartis Pharma, Pfizer, Sanofi, Sun Pharma, Taiho, Tanabe‐Mitsubishi, Torii, and UCB Pharma. A. Morita declares receiving research grants, consulting fees, and/or speaker’s fees from AbbVie, Boehringer Ingelheim, Celgene, Eli Lilly, Eisai, Janssen, Kyowa Hakko Kirin, LEO Pharma, Maruho, Mitsubishi Tanabe, Nichi‐Iko, Nippon Kayaku, Novartis, Sun Pharmaceutical Industries Taiho Pharmaceutical, Torii Pharmaceutical, and Ushio. N. Kotowsky, R. Gao, and K. Saito are employees of Boehringer Ingelheim.

## Supporting information

Supplementary Material

## Data Availability

We conducted a retrospective study using patient data from the JMDC database, a commercially available database of health claims and administrative data from health insurance associations. All patient data were anonymized and included no identifying information; therefore, informed consent was not necessary. Restrictions do apply to the availability of these data due to a contract between JMDC and Boehringer Ingelheim and are thus unavailable to the public. For inquiries on the database analyzed in this study, please contact JMDC (https://www.jmdc.co.jp/en/index).
