# Supplementary material for: Clinical characteristics and health‐care resource utilization in patients with generalized pustular psoriasis using real‐world evidence from the Japanese Medical Data Center database
Source: J Dermatol. 2021 Aug 13;48(11):1675–87. doi: 10.1111/1346-8138.16084 (PMC9290031; doi:10.1111/1346-8138.16084)
Supplement: Supplementary file 1 — Supplementary Material [file JDE-48-1675-s001.docx]

**Supplementary Materials**

**Title:** Clinical characteristics and health-care resource utilization in patients with generalized pustular psoriasis using real-world evidence from the Japanese Medical Data Center database

**Corresponding author:**

Ran Gao

Boehringer Ingelheim Pharmaceuticals Inc.

900 Ridgebury Road

Ridgefield

Connecticut 06877

USA

Email: [ran.gao@boehringer-ingelheim.com](mailto:ran.gao@boehringer-ingelheim.com)

Tel: (+1) 203-791-6660

**Content**

**Tables:**

Supplementary Table 1. List of comorbidities

Supplementary Table 2. List of dermatologic medications

Supplementary Table 3. List of medications for comorbidities

Supplementary Table 4. Added medications of interest

**Supplementary Table 1.** List of comorbidities

| **Comorbidity** | **ICD-10 code(s)†** |
| --- | --- |
| Allergies |  |
| Allergic rhinoconjunctivitis | J30.4 |
| Allergic contact dermatitis | L23 |
| Infections |  |
| Tonsillitis | Standard disease name |
| Sinusitis | Standard disease name |
| Autoimmune conditions |  |
| Psoriatic arthritis | L40.5 |
| Other psoriasis | L40.2, L40.4, L40.8, L40.9 |
| Pustulotic arthro-osteitis | Standard disease code: 8845161 |
| Dental conditions |  |
| Apical periodontal | Standard disease name |
| Periodontitis | Standard disease name |
| Alveolar pyorrhea | Standard disease name |
| Bone conditions |  |
| Osteoporosis | M80, M81 |
| Cardiovascular conditions |  |
| Myocardial infarction | I21.0, I21.1, I21.2, I21.3, I21.4, I21.9, I22.x |
| Stroke | I60.0, I60.1, I60.2, I60.3, I60.4, I60.5, I60.6, I60.7, I60.8, I60.9, I61.0, I61.1, I61.2, I61.3, I61.4, I61.5, I61.6, I61.8, I61.9, I62.0, I62.1, I62.9, I63.0, I63.1, I63.2, I63.3, I63.4, I63.5, I63.6, I63.8, I63.9 |
| Hypertension | I10.x – I15.x |
| Gastrointestinal conditions |  |
| Celiac disease | K90.0 |
| Crohn’s disease | K50.0, K50.1, K50.8, K50.9 |
| Diverticulitis | K57.0, K57.1, K57.2, K57.3, K57.4, K57.5, K57.8, K57.9 |
| Peptic ulcer disease | K25.0, K25.1, K25.2, K25.3, K25.4, K25.5, K25.6, K25.7, K25.9, K26.0, K26.1, K26.2, K26.3, K26.4, K26.5, K26.6, K26.7, K26.9, K27.0, K27.1, K27.2, K27.3, K27.4, K27.5, K27.6, K27.7, K27.9, K28.0, K28.1, K28.2, K28.3, K28.4, K28.5, K28.6, K28.7, K28.9 |
| Ulcerative colitis | K51.0, K51.2, K51.3, K51.4, K51.5, K51.8, K51.9 |
| Eye conditions |  |
| Uveitis | Standard disease name |
| Hepatic conditions |  |
| Non-alcoholic fatty liver disease | K76.0 |
| Hepatic failure | K72.0, K72.1, K72.9 |
| Hormonal/metabolic conditions |  |
| Type 2 diabetes | E11, E11.0, E11.1, E11.2, E11.3, E11.4, E11.5, E11.6, E11.7, E11.8, E11.9, E14, E14.0, E14.1, E14.2, E14.3, E14.4, E14.5, E14.6, E14.7, E14.8, E14.9  **AND** at least one antidiabetic treatment (insulin included) to occur within 30 days before or after the diagnosis code |
| Hyperlipidemia | E78.0, E78.1, E78.2, E78.3, E78.4, E78.5  **AND** at least one hyperlipidemia treatment |
| Metabolic syndrome | E88.8 |
| Obesity | E66.0, E66.3, E66.8, E66.9  **OR** BMI ≥25 kg/m^2^ calculated from height and weight |
| Hyperuricemia | Standard disease name |
| Thyroid disorders | Standard disease name |
| Pulmonary conditions |  |
| Asthma | J45.x |
| Chronic obstructive pulmonary disease | J40.x, J41.x, J42.x, J43.x, J44.x, J47.x |
| Interstitial pneumonia | Standard disease name |
| Psychiatric conditions |  |
| ADHD | F90.x |
| Anxiety | F06.4, F40.0, F40.1, F40.2, F40.8, F40.9, F41.0, F41.1, F41.2, F41.3, F41.8, F41.9, F43.2, F93.0 |
| Depression | F32, F33 |
| Schizophrenia | Standard disease name |
| Suicide ideation | **SDC codes:** 3009015, 3009016 |
| Sleep disorders |  |
| Sleep apnea | G47.3 |
| Insomnia | G47.0  **AND** at least one insomnia treatment within ±30 days |
| Renal conditions |  |
| Acute renal failure | N17.0, N17.1, N17.2, N17.8, N17.9, N19 |
| Chronic kidney disease | N18.1, N18.2, N18.3, N18.4, N18.5, N18.9 |
| Nephritic non-hypertensive disease | N11.0, N11.1. N11.8, N11.9, N12 |
| End-stage renal disease | N18.5, T82.4, T86.1, Z94.0 |
| Skin cancer |  |
| Basal cell carcinoma | SDC 1739003 |

†ICD-10 sub-codes will be added where applicable and appropriate.
ADHD, attention deficit hyperactivity disorder; BMI, body mass index; HTLV-1, human T-cell lymphotropic virus type 1; ICD-10 World Health Organization version, International Classification of Disease, 10th revision; SDC, standard disease code.

**Supplementary Table 2.** List of dermatologic medications

| **Class of medication for treatment of GPP or plaque psoriasis** | **Medications for treatment of GPP or plaque psoriasis** |
| --- | --- |
| Topical steroids | Plain, topical corticosteroids |
| Other topical medications | Benzoic acid/salicylic acid, lanolin, nystatin, dimethicone/petrolatum white, dimethicone, aloe vera/petrolatum hydrophilic, miconazole nitrate, tolnaftate, gentian violet, griseofulvin, clotrimazole, propylene glycol/mineral oil, ciclopirox olamine, ketoconazole, hydrophilic ointment, terbinafine hydrochloride, sertaconazole nitrate, econazole nitrate, ciclopirox, undecylenic acid, undecylenic acid/zinc undecylenate, sodium thiosulfate/salicylic acid, zinc oxide, vitamins and vitamin D/white petrolatum/lanolin, lanolin anhydrous, oxiconazole nitrate, efinaconazole, dimethicone/silicon dioxide, olive oil, tacrolimus, betamethasone/calcipotriene (Dovobet), maxacalcitol (Marduox)  **OR** ATC code: D05A (topical antipsoriasis products)  **AND** drug usage code = External medication |
| Systemic steroids (glucocorticoids) | ATC code: H02 |
| Tumor necrosis factor inhibitors | Adalimumab, certolizumab pegol, etanercept, infliximab, golimumab |
| Interleukin inhibitors | Ustekinumab, secukinumab, brodalumab, ixekizumab, guselkumab, risankizumab |
| Any biologic systemic | Adalimumab, certolizumab pegol, etanercept, infliximab, golimumab, ustekinumab, secukinumab, brodalumab, ixekizumab, guselkumab, abatacept, risankizumab |
| T-cell inhibitors | Abatacept |
| Phototherapy | Phototherapy |
| Any systemic (biologic or non-biologic) | Adalimumab, certolizumab pegol, etanercept, infliximab, golimumab, ustekinumab, secukinumab, brodalumab, ixekizumab, guselkumab, abatacept, risankizumab  **OR** etretinate, apremilast, vitamin D3, ciclosporine, hydroxycarbamide, methotrexate, mycophenolate mofetil, salazosulfapyridine, tacrolimus, tofacitinib, baricitinib, peficitinib, azathioprine  **OR** ATC code: H02  **AND** route not equal to ‘topical’ |
| Any systemic (non-biologic, any) | Etretinate, apremilast, vitamin D3, ciclosporine, hydroxycarbamide, methotrexate, mycophenolate mofetil, salazosulfapyridine, tacrolimus, tofacitinib, baricitinib, peficitinib, azathioprine  **OR** ATC code: H02  **AND** route not equal to ‘topical’ |

ATC, Anatomical Therapeutic Chemical; GPP, generalized pustular psoriasis.

**Supplementary Table 3.** List of medications for comorbidities

| **Class of medications for comorbidities** | **Medications for comorbidities** |
| --- | --- |
| Statins | Atorvastatin, fluvastatin, pravastatin, rosuvastatin, simvastatin, pitavastatin |
| Antihypertensive medication | Angiotensin-converting enzyme inhibitors, angiotensin receptor blockers, beta-blockers, calcium channel blockers, diuretics, alpha-blockers, alpha-beta-blockers |
| Antibiotics | Penicillins, cephalosporins, macrolides, fluoroquinolones, sulfonamides, tetracyclines, aminoglycosides |
| Type 2 diabetes medication | ATC code: A10 – Drugs used in diabetes |
| Asthma medication | Fluticasone, budesonide, mometasone, beclometasone, ciclesonide, flunisolide, montelukast, pranlukast hydrate, zafirlukast, indacaterol maleate, salmeterol, formoterol, fluticasone and salmeterol, budesonide and formoterol, umeclidinium bromide/vilanterol trifenatate, vilanterol trifenatate/fluticasone furoate, fluticasone furoate/umeclidinium bromide/vilanterol trifenatate, salbutamol sulfate, ipratropium, prednisolone, omalizumab, mepolizumab, benralizumab |
| Chronic obstructive pulmonary disease medication | Salbutamol sulfate, ipratropium, fluticasone, budesonide, prednisolone (nasal), aclidinium, formoterol, glycopyrronium bromide, indacaterol, olodaterol, salmeterol, tiotropium, umeclidinium, glycopyrronium/formoterol, glycopyrronium/indacaterol, tiotropium/olodaterol, umeclidinium/vilanterol, budesonide/formoterol, fluticasone/salmeterol, fluticasone/vilanterol |
| Psychiatric medication | Antipsychotics  Hypnotics and sedatives  Antidepressants  Tranquilizers |
| Opioid pain medication | ATC codes – N02AJ, N02AX |
| Sleep medication (non-benzodiazepine sedatives) | Eszopiclone, zolpidem |
| Benzodiazepine sedatives | Triazolam, estazolam, lorazepam, flurazepam, quazepam |
| Hyperlipidemia medication | HMG-CoA reductase inhibitors (statins): atorvastatin, fluvastatin, pravastatin, rosuvastatin, simvastatin, pitavastatin  Intestinal cholesterol transporter inhibitor: ezetimibe  Resins: colestimide and cholestyramine probucol  PCSK9 inhibitors: evolocumab, alirocumab  MTP inhibitor: lomitapide  Fibrates: bezafibrate, fenofibrate, clinofibrate, clofibrate  SPPARM alpha: pemafibrate  Nicotinic acid derivatives: niceritrol, nicomol, tocopheryl nicotinate  PUFAs: ethyl icosapentate and omega-3-acid-ethyl ester |

ATC, Anatomical Therapeutic Chemical; HMG-CoA, hydroxymethylglutaryl-Coenzyme A; MTP, microsomal triglyceride transfer protein; PCSK9, proprotein convertase subtilisin/kexin type 9; PUFA, polyunsaturated fatty acids, SSPARM, selective peroxisome proliferator alpha modulator.

**Supplementary Table 4.** Added medications of interest

| **Added medications of interest** | **Search term** |
| --- | --- |
| Etretinate | Etretinate |
| Adalimumab | Adalimumab |
| Apremilast | Apremilast |
| Brodalumab | Brodalumab |
| Certolizumab | Certolizumab pegol |
| Ciclosporine | Ciclosporine |
| Etanercept | Etanercept |
| Golimumab | Golimumab |
| Guselkumab | Guselkumab |
| Infliximab | Infliximab, infliximab-abda, infliximab-dyyb |
| Ixekizumab | Ixekizumab |
| Methotrexate | Methotrexate, methotrexate sodium, methotrexate sodium/pf, methotrexate/pf |
| Retinoic acid | Tretinoin retinol palmitate |
| Secukinumab | Secukinumab |
| Tofacitinib | Tofacitinib citrate |
| Ustekinumab | Ustekinumab |
| Risankizumab | Risankizumab |
| Vitamin D3 | Vitamin D3 topical |
| Dovobet | Calcipotriol hydrate + betamethasone dipropionate |
| Maxacalcitol | Maxacalcitol (exclude Marduox) |
| Marduox | Betamethasone butyrate propionate |
| Azathioprine | Azathioprine |
| Tacrolimus | Prograf, graceptor, tacrolimus (oral or injection or topical) |
| Adacolumn (GMA, apheresis) | Standard material code: plas/732030000 |
| Plasma exchange | Medication/procedure/material name |

GMA, granulocyte/monocyte adsorption.
